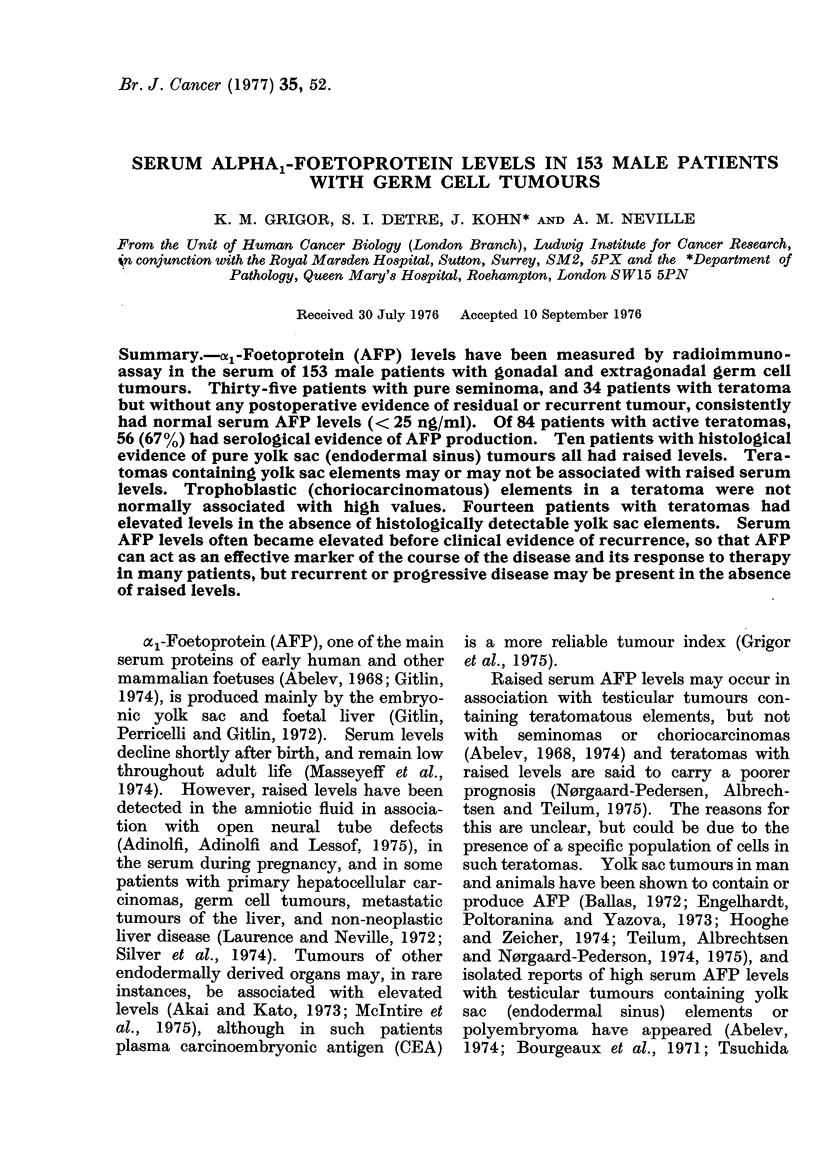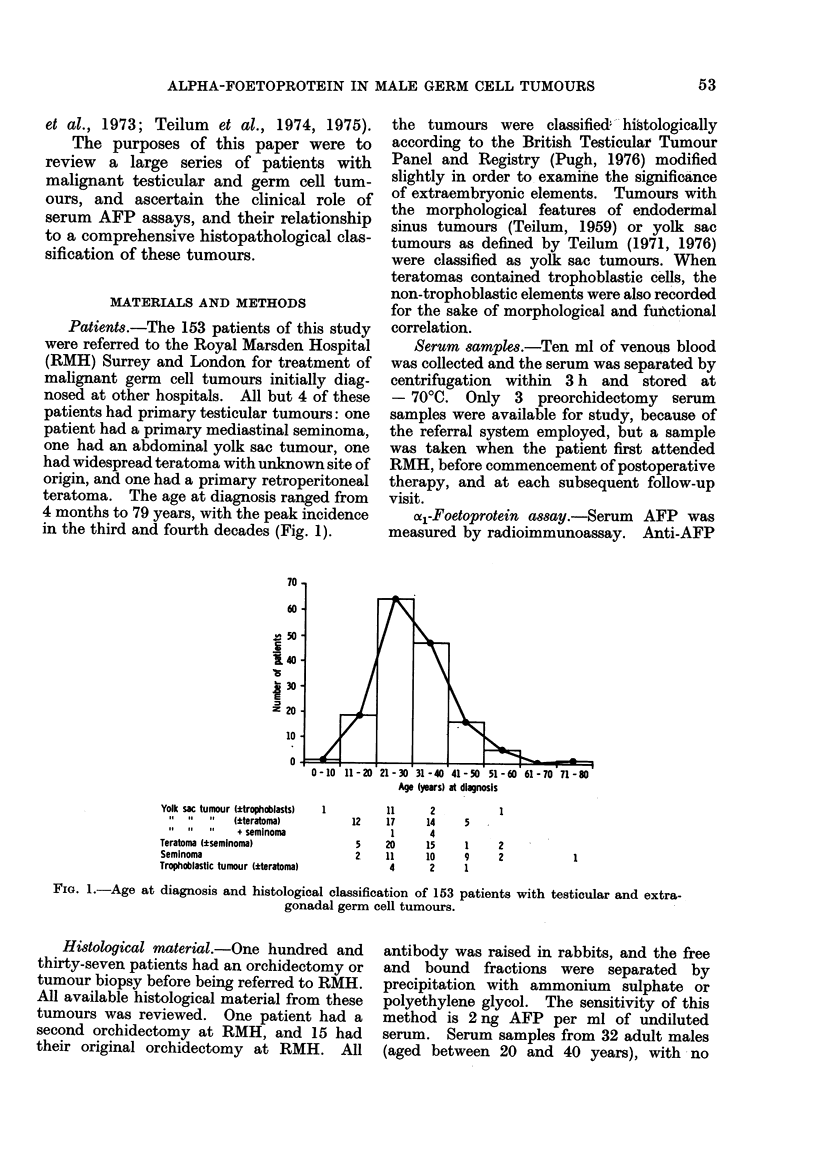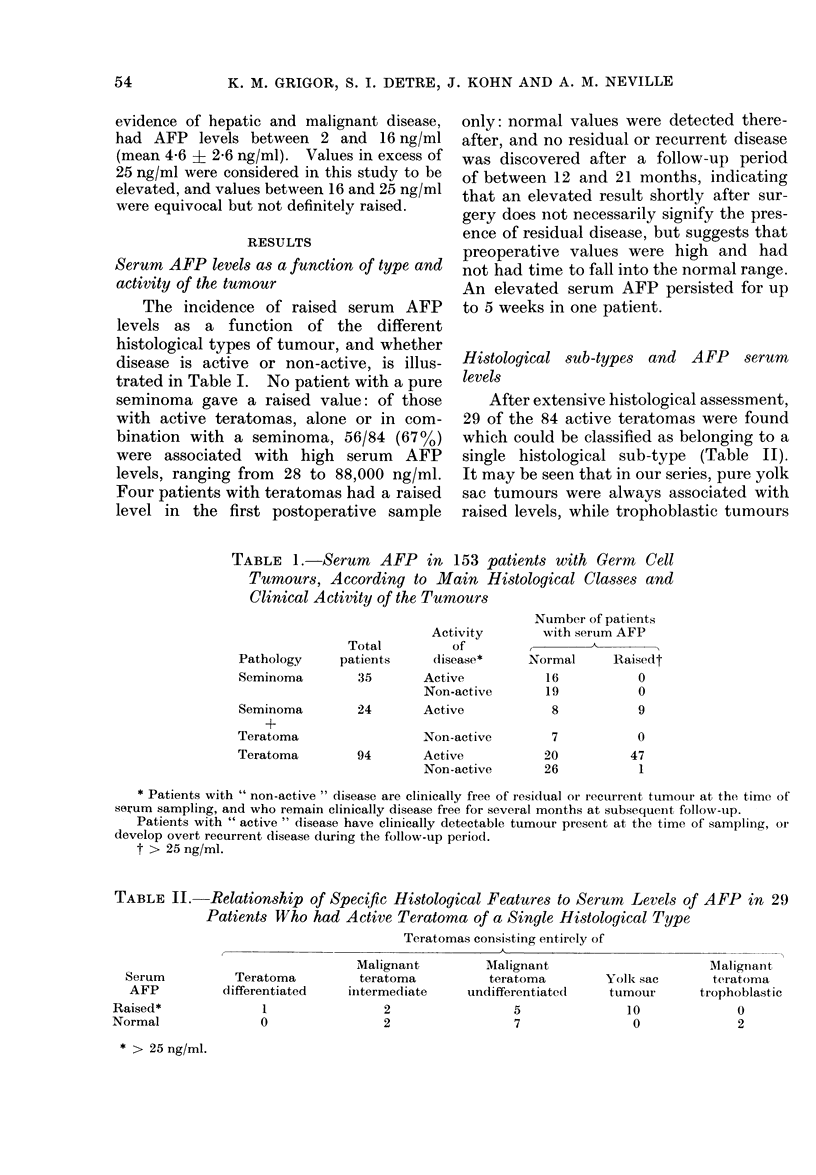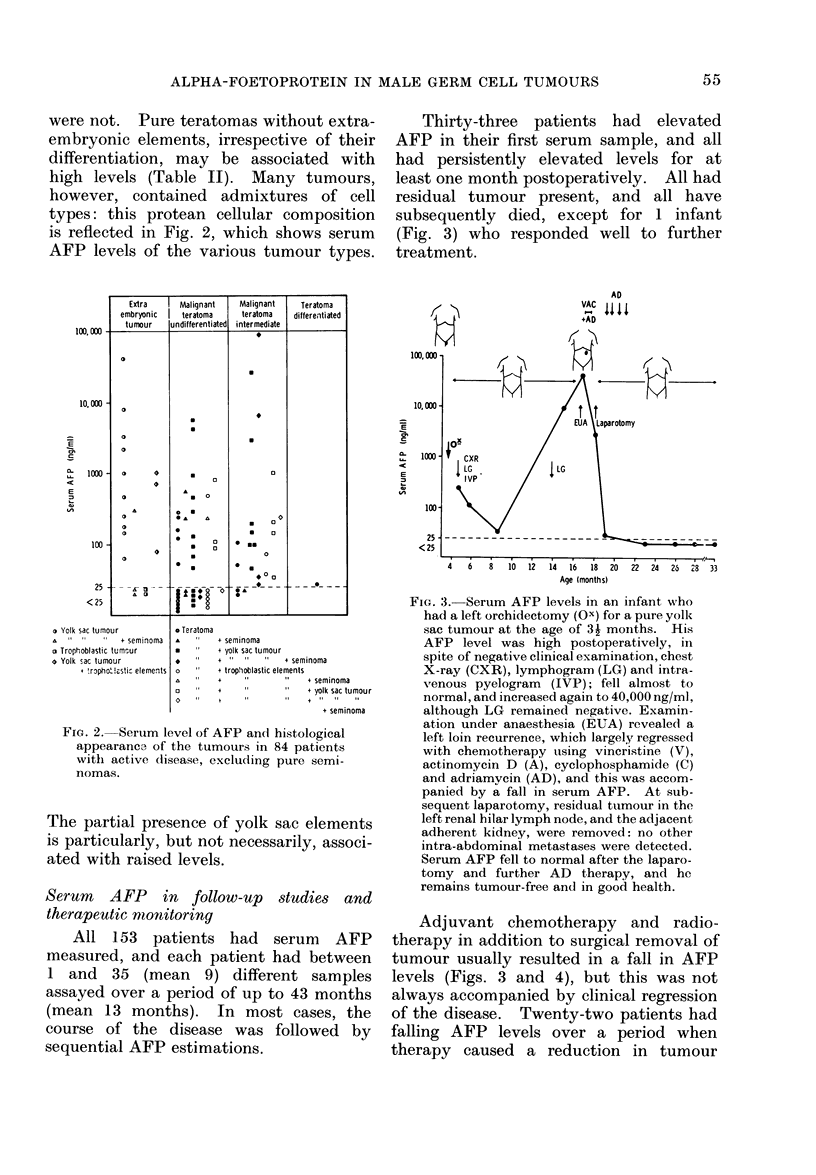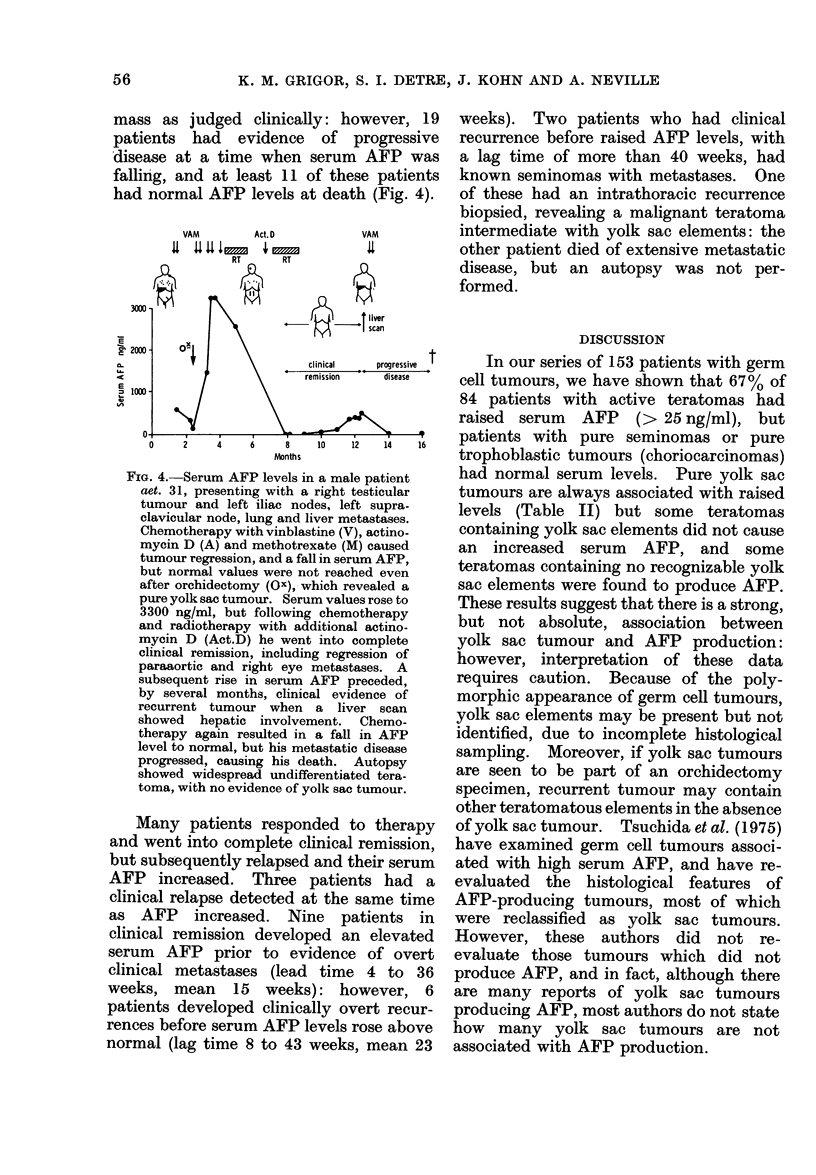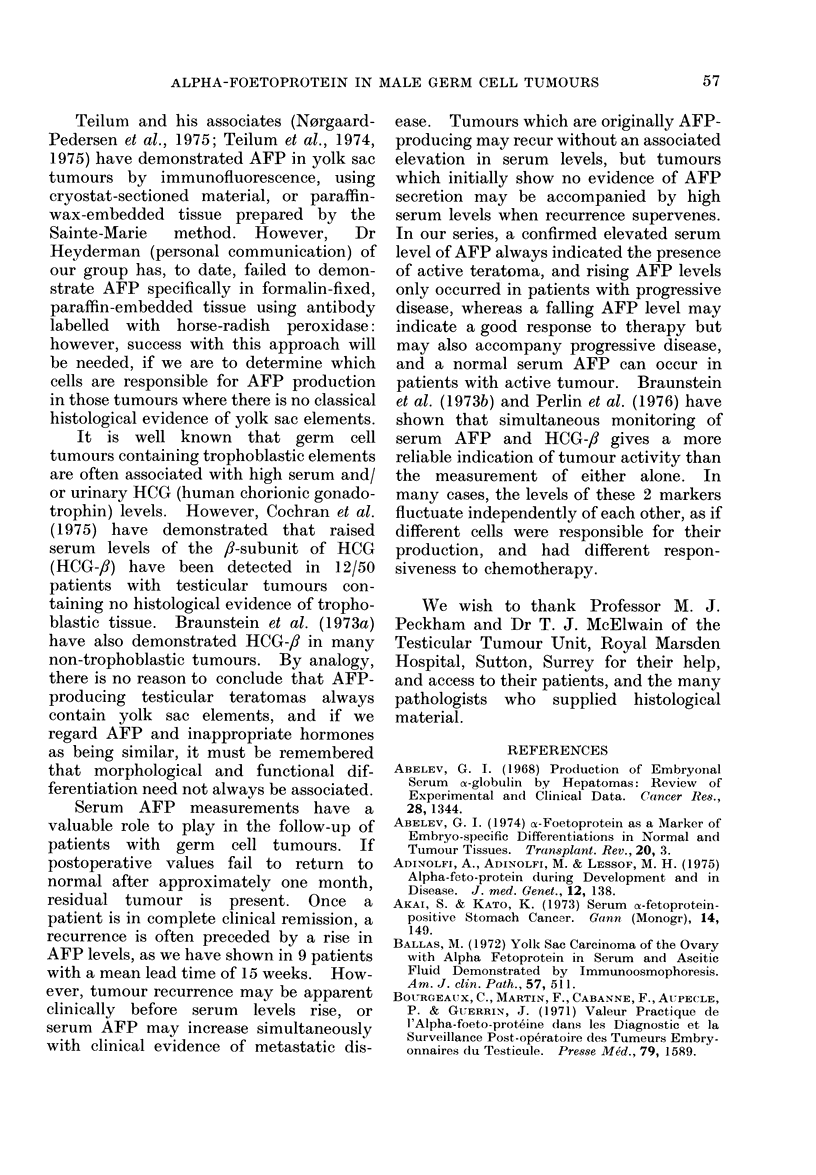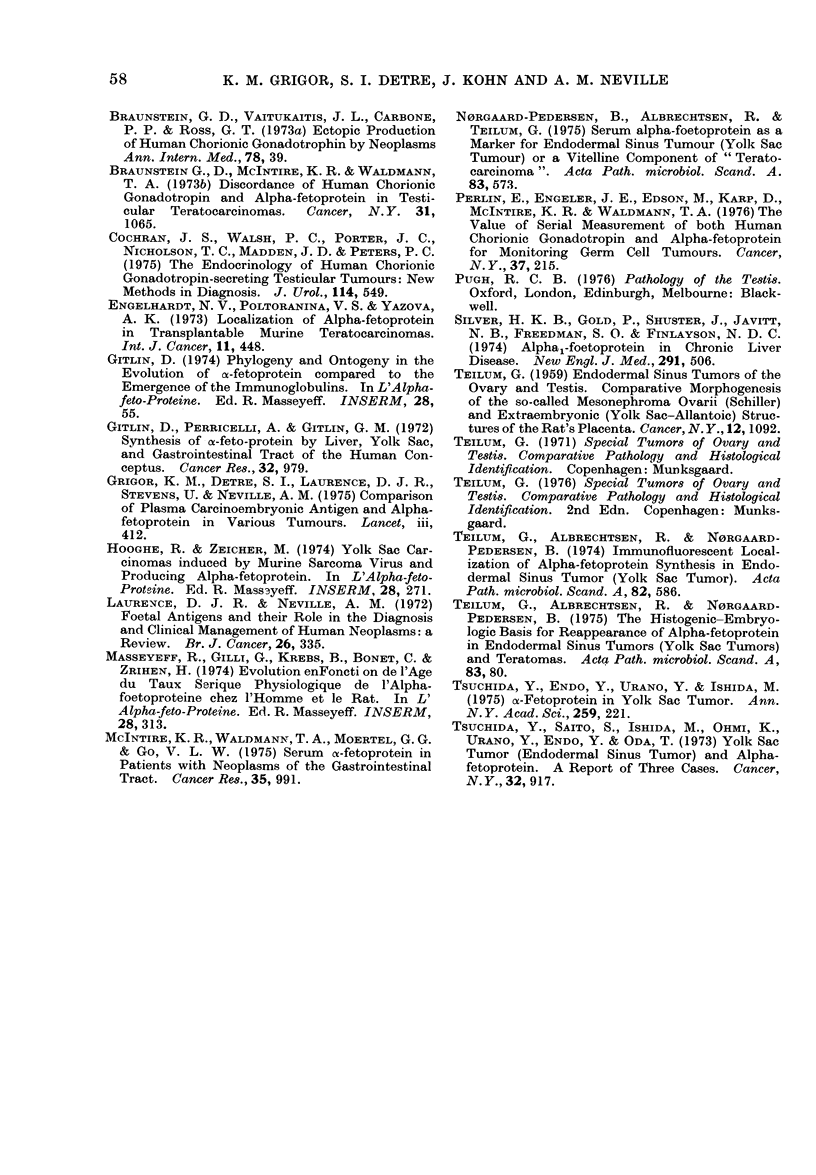# Serum alpha1-foetoprotein levels in 153 male patients with germ cell tumours.

**DOI:** 10.1038/bjc.1977.3

**Published:** 1977-01

**Authors:** K. M. Grigor, S. I. Detre, J. Kohn, A. M. Neville

## Abstract

--alpha1-Foetoprotein (AFP) levels have been measured by radioimmunoassay in the serum of 153 male patients with gonadal and extragonadal germ cell tumours. Thirty-five patients with pure seminoma, and 34 patients with teratoma but without any postoperative evidence of residual or recurrent tumour, consistently had normal serum AFP levels (less than 25 ng/ml). Of 84 patients with active teratomas, 56 (67%) had serological evidence of AFP production. Ten patients with histological evidence of pure yolk sac (endodermal sinus) tumours all had raised levels. Teratomas containing yolk sac (elements may or may not be associated with raised serum levels. Trophoblastic (choriocarcinomatous) elements in a teratoma were not normally associated with high values. Fourteen patients with teratomas had elevated levels in the absence of histologically detectable yolk sac elements. Serum AFP levels often became elevated before clinical evidence of recurrence, so that AFP can act as an effective marker of the course of the disease and its response to therapy in many patients, but recurrent or progressive disease may be present in the absence of raised levels.


*Br. J. Cancer* (1977) **35**, 52.

### SERUM ALPHA$_1$-FOETOPROTEIN LEVELS IN 153 MALE PATIENTS WITH GERM CELL TUMOURS

K. M. GRIGOR, S. I. DETRE, J. KOHN* AND A. M. NEVILLE

*From the Unit of Human Cancer Biology (London Branch), Ludwig Institute for Cancer Research, in conjunction with the Royal Marsden Hospital, Sutton, Surrey, SM2, 5PX and the *Department of Pathology, Queen Mary's Hospital, Roehampton, London SW15 5PN*

Received 30 July 1976 Accepted 10 September 1976

**Summary.—$\alpha_1$-Foetoprotein (AFP) levels have been measured by radioimmunoassay in the serum of 153 male patients with gonadal and extragonadal germ cell tumours. Thirty-five patients with pure seminoma, and 34 patients with teratoma but without any postoperative evidence of residual or recurrent tumour, consistently had normal serum AFP levels ($<$ 25 ng/ml). Of 84 patients with active teratomas, 56 (67%) had serological evidence of AFP production. Ten patients with histological evidence of pure yolk sac (endodermal sinus) tumours all had raised levels. Teratomas containing yolk sac elements may or may not be associated with raised serum levels. Trophoblastic (choriocarcinomatous) elements in a teratoma were not normally associated with high values. Fourteen patients with teratomas had elevated levels in the absence of histologically detectable yolk sac elements. Serum AFP levels often became elevated before clinical evidence of recurrence, so that AFP can act as an effective marker of the course of the disease and its response to therapy in many patients, but recurrent or progressive disease may be present in the absence of raised levels.**

$\alpha_1$-Foetoprotein (AFP), one of the main serum proteins of early human and other mammalian foetuses (Abelev, 1968; Gitlin, 1974), is produced mainly by the embryonic yolk sac and foetal liver (Gitlin, Perricelli and Gitlin, 1972). Serum levels decline shortly after birth, and remain low throughout adult life (Masseyeff *et al.*, 1974). However, raised levels have been detected in the amniotic fluid in association with open neural tube defects (Adinolfi, Adinolfi and Lessof, 1975), in the serum during pregnancy, and in some patients with primary hepatocellular carcinomas, germ cell tumours, metastatic tumours of the liver, and non-neoplastic liver disease (Laurence and Neville, 1972; Silver *et al.*, 1974). Tumours of other endodermally derived organs may, in rare instances, be associated with elevated levels (Akai and Kato, 1973; McIntire *et al.*, 1975), although in such patients plasma carcinoembryonic antigen (CEA) is a more reliable tumour index (Grigor *et al.*, 1975).

Raised serum AFP levels may occur in association with testicular tumours containing teratomatous elements, but not with seminomas or choriocarcinomas (Abelev, 1968, 1974) and teratomas with raised levels are said to carry a poorer prognosis (Nørgaard-Pedersen, Albrechtsen and Teilum, 1975). The reasons for this are unclear, but could be due to the presence of a specific population of cells in such teratomas. Yolk sac tumours in man and animals have been shown to contain or produce AFP (Ballas, 1972; Engelhardt, Poltoranina and Yazova, 1973; Hooghe and Zeicher, 1974; Teilum, Albrechtsen and Nørgaard-Pederson, 1974, 1975), and isolated reports of high serum AFP levels with testicular tumours containing yolk sac (endodermal sinus) elements or polyembryoma have appeared (Abelev, 1974; Bourgeaux *et al.*, 1971; Tsuchida

*et al.*, 1973; Teilum *et al.*, 1974, 1975).

The purposes of this paper were to review a large series of patients with malignant testicular and germ cell tumours, and ascertain the clinical role of serum AFP assays, and their relationship to a comprehensive histopathological classification of these tumours.

### MATERIALS AND METHODS

*Patients.*—The 153 patients of this study were referred to the Royal Marsden Hospital (RMH) Surrey and London for treatment of malignant germ cell tumours initially diagnosed at other hospitals. All but 4 of these patients had primary testicular tumours: one patient had a primary mediastinal seminoma, one had an abdominal yolk sac tumour, one had widespread teratoma with unknown site of origin, and one had a primary retroperitoneal teratoma. The age at diagnosis ranged from 4 months to 79 years, with the peak incidence in the third and fourth decades (Fig. 1).

the tumours were classified histologically according to the British Testicular Tumour Panel and Registry (Pugh, 1976) modified slightly in order to examine the significance of extraembryonic elements. Tumours with the morphological features of endodermal sinus tumours (Teilum, 1959) or yolk sac tumours as defined by Teilum (1971, 1976) were classified as yolk sac tumours. When teratomas contained trophoblastic cells, the non-trophoblastic elements were also recorded for the sake of morphological and functional correlation.

*Serum samples.*—Ten ml of venous blood was collected and the serum was separated by centrifugation within 3 h and stored at − 70°C. Only 3 preorchidectomy serum samples were available for study, because of the referral system employed, but a sample was taken when the patient first attended RMH, before commencement of postoperative therapy, and at each subsequent follow-up visit.

*$\alpha_1$-Foetoprotein assay.*—Serum AFP was measured by radioimmunoassay. Anti-AFP

| | 0-10 | 11-20 | 21-30 | 31-40 | 41-50 | 51-60 | 61-70 | 71-80 |
|---|---|---|---|---|---|---|---|---|
| Yolk sac tumour (±trophoblasts) | 1 | | 11 | 2 | | 1 | | |
| " " " (±teratoma) | | 12 | 17 | 14 | 5 | | | |
| " " " + seminoma | | | 1 | 4 | | | | |
| Teratoma (±seminoma) | | 5 | 20 | 15 | 1 | 2 | | |
| Seminoma | | 2 | 11 | 10 | 9 | 2 | | 1 |
| Trophoblastic tumour (±teratoma) | | | 4 | 2 | 1 | | | |

FIG. 1.—Age at diagnosis and histological classification of 153 patients with testicular and extragonadal germ cell tumours.

*Histological material.*—One hundred and thirty-seven patients had an orchidectomy or tumour biopsy before being referred to RMH. All available histological material from these tumours was reviewed. One patient had a second orchidectomy at RMH, and 15 had their original orchidectomy at RMH. All antibody was raised in rabbits, and the free and bound fractions were separated by precipitation with ammonium sulphate or polyethylene glycol. The sensitivity of this method is 2 ng AFP per ml of undiluted serum. Serum samples from 32 adult males (aged between 20 and 40 years), with no evidence of hepatic and malignant disease, had AFP levels between 2 and 16 ng/ml (mean 4·6 ± 2·6 ng/ml). Values in excess of 25 ng/ml were considered in this study to be elevated, and values between 16 and 25 ng/ml were equivocal but not definitely raised.

### RESULTS

### *Serum AFP levels as a function of type and activity of the tumour*

The incidence of raised serum AFP levels as a function of the different histological types of tumour, and whether disease is active or non-active, is illustrated in Table I. No patient with a pure seminoma gave a raised value: of those with active teratomas, alone or in combination with a seminoma, 56/84 (67%) were associated with high serum AFP levels, ranging from 28 to 88,000 ng/ml. Four patients with teratomas had a raised level in the first postoperative sample only: normal values were detected thereafter, and no residual or recurrent disease was discovered after a follow-up period of between 12 and 21 months, indicating that an elevated result shortly after surgery does not necessarily signify the presence of residual disease, but suggests that preoperative values were high and had not had time to fall into the normal range. An elevated serum AFP persisted for up to 5 weeks in one patient.

### *Histological sub-types and AFP serum levels*

After extensive histological assessment, 29 of the 84 active teratomas were found which could be classified as belonging to a single histological sub-type (Table II). It may be seen that in our series, pure yolk sac tumours were always associated with raised levels, while trophoblastic tumours

TABLE I.—*Serum AFP in 153 patients with Germ Cell Tumours, According to Main Histological Classes and Clinical Activity of the Tumours*

| Pathology | Total patients | Activity of disease* | Number of patients with serum AFP: Normal | Raised† |
|---|---|---|---|---|
| Seminoma | 35 | Active | 16 | 0 |
| | | Non-active | 19 | 0 |
| Seminoma + Teratoma | 24 | Active | 8 | 9 |
| | | Non-active | 7 | 0 |
| Teratoma | 94 | Active | 20 | 47 |
| | | Non-active | 26 | 1 |

* Patients with " non-active " disease are clinically free of residual or recurrent tumour at the time of serum sampling, and who remain clinically disease free for several months at subsequent follow-up.

Patients with " active " disease have clinically detectable tumour present at the time of sampling, or develop overt recurrent disease during the follow-up period.

† > 25 ng/ml.

TABLE II.—*Relationship of Specific Histological Features to Serum Levels of AFP in 29 Patients Who had Active Teratoma of a Single Histological Type*

| Serum AFP | Teratomas consisting entirely of: Teratoma differentiated | Malignant teratoma intermediate | Malignant teratoma undifferentiated | Yolk sac tumour | Malignant teratoma trophoblastic |
|---|---|---|---|---|---|
| Raised* | 1 | 2 | 5 | 10 | 0 |
| Normal | 0 | 2 | 7 | 0 | 2 |

* > 25 ng/ml.

were not. Pure teratomas without extra-embryonic elements, irrespective of their differentiation, may be associated with high levels (Table II). Many tumours, however, contained admixtures of cell types: this protean cellular composition is reflected in Fig. 2, which shows serum AFP levels of the various tumour types.

Fig. 2.—Serum level of AFP and histological appearance of the tumours in 84 patients with active disease, excluding pure seminomas.

The partial presence of yolk sac elements is particularly, but not necessarily, associated with raised levels.

*Serum AFP in follow-up studies and therapeutic monitoring*

All 153 patients had serum AFP measured, and each patient had between 1 and 35 (mean 9) different samples assayed over a period of up to 43 months (mean 13 months). In most cases, the course of the disease was followed by sequential AFP estimations.

Thirty-three patients had elevated AFP in their first serum sample, and all had persistently elevated levels for at least one month postoperatively. All had residual tumour present, and all have subsequently died, except for 1 infant (Fig. 3) who responded well to further treatment.

Fig. 3.—Serum AFP levels in an infant who had a left orchidectomy ($O^{x}$) for a pure yolk sac tumour at the age of $3\frac{1}{2}$ months. His AFP level was high postoperatively, in spite of negative clinical examination, chest X-ray (CXR), lymphogram (LG) and intravenous pyelogram (IVP); fell almost to normal, and increased again to 40,000 ng/ml, although LG remained negative. Examination under anaesthesia (EUA) revealed a left loin recurrence, which largely regressed with chemotherapy using vincristine (V), actinomycin D (A), cyclophosphamide (C) and adriamycin (AD), and this was accompanied by a fall in serum AFP. At subsequent laparotomy, residual tumour in the left renal hilar lymph node, and the adjacent adherent kidney, were removed: no other intra-abdominal metastases were detected. Serum AFP fell to normal after the laparotomy and further AD therapy, and he remains tumour-free and in good health.

Adjuvant chemotherapy and radiotherapy in addition to surgical removal of tumour usually resulted in a fall in AFP levels (Figs. 3 and 4), but this was not always accompanied by clinical regression of the disease. Twenty-two patients had falling AFP levels over a period when therapy caused a reduction in tumour mass as judged clinically: however, 19 patients had evidence of progressive disease at a time when serum AFP was falling, and at least 11 of these patients had normal AFP levels at death (Fig. 4).

FIG. 4.—Serum AFP levels in a male patient *aet.* 31, presenting with a right testicular tumour and left iliac nodes, left supraclavicular node, lung and liver metastases. Chemotherapy with vinblastine (V), actinomycin D (A) and methotrexate (M) caused tumour regression, and a fall in serum AFP, but normal values were not reached even after orchidectomy ($O^x$), which revealed a pure yolk sac tumour. Serum values rose to 3300 ng/ml, but following chemotherapy and radiotherapy with additional actinomycin D (Act.D) he went into complete clinical remission, including regression of paraaortic and right eye metastases. A subsequent rise in serum AFP preceded, by several months, clinical evidence of recurrent tumour when a liver scan showed hepatic involvement. Chemotherapy again resulted in a fall in AFP level to normal, but his metastatic disease progressed, causing his death. Autopsy showed widespread undifferentiated teratoma, with no evidence of yolk sac tumour.

Many patients responded to therapy and went into complete clinical remission, but subsequently relapsed and their serum AFP increased. Three patients had a clinical relapse detected at the same time as AFP increased. Nine patients in clinical remission developed an elevated serum AFP prior to evidence of overt clinical metastases (lead time 4 to 36 weeks, mean 15 weeks): however, 6 patients developed clinically overt recurrences before serum AFP levels rose above normal (lag time 8 to 43 weeks, mean 23 weeks). Two patients who had clinical recurrence before raised AFP levels, with a lag time of more than 40 weeks, had known seminomas with metastases. One of these had an intrathoracic recurrence biopsied, revealing a malignant teratoma intermediate with yolk sac elements: the other patient died of extensive metastatic disease, but an autopsy was not performed.

### DISCUSSION

In our series of 153 patients with germ cell tumours, we have shown that 67% of 84 patients with active teratomas had raised serum AFP (> 25 ng/ml), but patients with pure seminomas or pure trophoblastic tumours (choriocarcinomas) had normal serum levels. Pure yolk sac tumours are always associated with raised levels (Table II) but some teratomas containing yolk sac elements did not cause an increased serum AFP, and some teratomas containing no recognizable yolk sac elements were found to produce AFP. These results suggest that there is a strong, but not absolute, association between yolk sac tumour and AFP production: however, interpretation of these data requires caution. Because of the polymorphic appearance of germ cell tumours, yolk sac elements may be present but not identified, due to incomplete histological sampling. Moreover, if yolk sac tumours are seen to be part of an orchidectomy specimen, recurrent tumour may contain other teratomatous elements in the absence of yolk sac tumour. Tsuchida *et al.* (1975) have examined germ cell tumours associated with high serum AFP, and have re-evaluated the histological features of AFP-producing tumours, most of which were reclassified as yolk sac tumours. However, these authors did not re-evaluate those tumours which did not produce AFP, and in fact, although there are many reports of yolk sac tumours producing AFP, most authors do not state how many yolk sac tumours are not associated with AFP production.

Teilum and his associates (Nørgaard-Pedersen *et al.*, 1975; Teilum *et al.*, 1974, 1975) have demonstrated AFP in yolk sac tumours by immunofluorescence, using cryostat-sectioned material, or paraffin-wax-embedded tissue prepared by the Sainte-Marie method. However, Dr Heyderman (personal communication) of our group has, to date, failed to demonstrate AFP specifically in formalin-fixed, paraffin-embedded tissue using antibody labelled with horse-radish peroxidase: however, success with this approach will be needed, if we are to determine which cells are responsible for AFP production in those tumours where there is no classical histological evidence of yolk sac elements.

It is well known that germ cell tumours containing trophoblastic elements are often associated with high serum and/or urinary HCG (human chorionic gonadotrophin) levels. However, Cochran *et al.* (1975) have demonstrated that raised serum levels of the $\beta$-subunit of HCG (HCG-$\beta$) have been detected in 12/50 patients with testicular tumours containing no histological evidence of trophoblastic tissue. Braunstein *et al.* (1973*a*) have also demonstrated HCG-$\beta$ in many non-trophoblastic tumours. By analogy, there is no reason to conclude that AFP-producing testicular teratomas always contain yolk sac elements, and if we regard AFP and inappropriate hormones as being similar, it must be remembered that morphological and functional differentiation need not always be associated.

Serum AFP measurements have a valuable role to play in the follow-up of patients with germ cell tumours. If postoperative values fail to return to normal after approximately one month, residual tumour is present. Once a patient is in complete clinical remission, a recurrence is often preceded by a rise in AFP levels, as we have shown in 9 patients with a mean lead time of 15 weeks. However, tumour recurrence may be apparent clinically before serum levels rise, or serum AFP may increase simultaneously with clinical evidence of metastatic disease. Tumours which are originally AFP-producing may recur without an associated elevation in serum levels, but tumours which initially show no evidence of AFP secretion may be accompanied by high serum levels when recurrence supervenes. In our series, a confirmed elevated serum level of AFP always indicated the presence of active teratoma, and rising AFP levels only occurred in patients with progressive disease, whereas a falling AFP level may indicate a good response to therapy but may also accompany progressive disease, and a normal serum AFP can occur in patients with active tumour. Braunstein *et al.* (1973*b*) and Perlin *et al.* (1976) have shown that simultaneous monitoring of serum AFP and HCG-$\beta$ gives a more reliable indication of tumour activity than the measurement of either alone. In many cases, the levels of these 2 markers fluctuate independently of each other, as if different cells were responsible for their production, and had different responsiveness to chemotherapy.

We wish to thank Professor M. J. Peckham and Dr T. J. McElwain of the Testicular Tumour Unit, Royal Marsden Hospital, Sutton, Surrey for their help, and access to their patients, and the many pathologists who supplied histological material.

REFERENCES

Abelev, G. I. (1968) Production of Embryonal Serum $\alpha$-globulin by Hepatomas: Review of Experimental and Clinical Data. *Cancer Res.*, **28**, 1344.

Abelev, G. I. (1974) $\alpha$-Foetoprotein as a Marker of Embryo-specific Differentiations in Normal and Tumour Tissues. *Transplant. Rev.*, **20**, 3.

Adinolfi, A., Adinolfi, M. & Lessof, M. H. (1975) Alpha-feto-protein during Development and in Disease. *J. med. Genet.*, **12**, 138.

Akai, S. & Kato, K. (1973) Serum $\alpha$-fetoprotein-positive Stomach Cancer. *Gann* (Monogr), **14**, 149.

Ballas, M. (1972) Yolk Sac Carcinoma of the Ovary with Alpha Fetoprotein in Serum and Ascitic Fluid Demonstrated by Immunoosmophoresis. *Am. J. clin. Path.*, **57**, 511.

Bourgeaux, C., Martin, F., Cabanne, F., Aupecle, P. & Guerrin, J. (1971) Valeur Practique de l'Alpha-foeto-protéine dans les Diagnostic et la Surveillance Post-opératoire des Tumeurs Embryonnaires du Testicule. *Presse Méd.*, **79**, 1589.

BRAUNSTEIN, G. D., VAITUKAITIS, J. L., CARBONE, P. P. & ROSS, G. T. (1973*a*) Ectopic Production of Human Chorionic Gonadotrophin by Neoplasms *Ann. Intern. Med.*, **78**, 39.

BRAUNSTEIN G., D., McINTIRE, K. R. & WALDMANN, T. A. (1973*b*) Discordance of Human Chorionic Gonadotropin and Alpha-fetoprotein in Testicular Teratocarcinomas. *Cancer, N.Y.* **31**, 1065.

COCHRAN, J. S., WALSH, P. C., PORTER, J. C., NICHOLSON, T. C., MADDEN, J. D. & PETERS, P. C. (1975) The Endocrinology of Human Chorionic Gonadotropin-secreting Testicular Tumours: New Methods in Diagnosis. *J. Urol.*, **114**, 549.

ENGELHARDT, N. V., POLTORANINA, V. S. & YAZOVA, A. K. (1973) Localization of Alpha-fetoprotein in Transplantable Murine Teratocarcinomas. *Int. J. Cancer*, **11**, 448.

GITLIN, D. (1974) Phylogeny and Ontogeny in the Evolution of α-fetoprotein compared to the Emergence of the Immunoglobulins. In *L'Alpha-feto-Proteine*. Ed. R. Masseyeff. *INSERM*, **28**, 55.

GITLIN, D., PERRICELLI, A. & GITLIN, G. M. (1972) Synthesis of α-feto-protein by Liver, Yolk Sac, and Gastrointestinal Tract of the Human Conceptus. *Cancer Res.*, **32**, 979.

GRIGOR, K. M., DETRE, S. I., LAURENCE, D. J. R., STEVENS, U. & NEVILLE, A. M. (1975) Comparison of Plasma Carcinoembryonic Antigen and Alpha-fetoprotein in Various Tumours. *Lancet*, iii, 412.

HOOGHE, R. & ZEICHER, M. (1974) Yolk Sac Carcinomas induced by Murine Sarcoma Virus and Producing Alpha-fetoprotein. In *L'Alpha-feto-Proteine*. Ed. R. Masseyeff. *INSERM*, **28**, 271.

LAURENCE, D. J. R. & NEVILLE, A. M. (1972) Foetal Antigens and their Role in the Diagnosis and Clinical Management of Human Neoplasms: a Review. *Br. J. Cancer*, **26**, 335.

MASSEYEFF, R., GILLI, G., KREBS, B., BONET, C. & ZRIHEN, H. (1974) Evolution enFoncti on de l'Age du Taux Serique Physiologique de l'Alpha-foetoproteine chez l'Homme et le Rat. In *L' Alpha-feto-Proteine*. Ed. R. Masseyeff. *INSERM*, **28**, 313.

McINTIRE, K. R., WALDMANN, T. A., MOERTEL, G. G. & GO, V. L. W. (1975) Serum α-fetoprotein in Patients with Neoplasms of the Gastrointestinal Tract. *Cancer Res.*, **35**, 991.

NØRGAARD-PEDERSEN, B., ALBRECHTSEN, R. & TEILUM, G. (1975) Serum alpha-foetoprotein as a Marker for Endodermal Sinus Tumour (Yolk Sac Tumour) or a Vitelline Component of "Teratocarcinoma". *Acta Path. microbiol. Scand. A*. **83**, 573.

PERLIN, E., ENGELER, J. E., EDSON, M., KARP, D., McINTIRE, K. R. & WALDMANN, T. A. (1976) The Value of Serial Measurement of both Human Chorionic Gonadotropin and Alpha-fetoprotein for Monitoring Germ Cell Tumours. *Cancer, N.Y.*, **37**, 215.

PUGH, R. C. B. (1976) *Pathology of the Testis*. Oxford, London, Edinburgh, Melbourne: Blackwell.

SILVER, H. K. B., GOLD, P., SHUSTER, J., JAVITT, N. B., FREEDMAN, S. O. & FINLAYSON, N. D. C. (1974) Alpha$_1$-foetoprotein in Chronic Liver Disease. *New Engl. J. Med.*, **291**, 506.

TEILUM, G. (1959) Endodermal Sinus Tumors of the Ovary and Testis. Comparative Morphogenesis of the so-called Mesonephroma Ovarii (Schiller) and Extraembryonic (Yolk Sac–Allantoic) Structures of the Rat's Placenta. *Cancer, N.Y.*, **12**, 1092.

TEILUM, G. (1971) *Special Tumors of Ovary and Testis. Comparative Pathology and Histological Identification*. Copenhagen: Munksgaard.

TEILUM, G. (1976) *Special Tumors of Ovary and Testis. Comparative Pathology and Histological Identification*. 2nd Edn. Copenhagen: Munksgaard.

TEILUM, G., ALBRECHTSEN, R. & NØRGAARD-PEDERSEN, B. (1974) Immunofluorescent Localization of Alpha-fetoprotein Synthesis in Endodermal Sinus Tumor (Yolk Sac Tumor). *Acta Path. microbiol. Scand. A*, **82**, 586.

TEILUM, G., ALBRECHTSEN, R. & NØRGAARD-PEDERSEN, B. (1975) The Histogenic–Embryologic Basis for Reappearance of Alpha-fetoprotein in Endodermal Sinus Tumors (Yolk Sac Tumors) and Teratomas. *Acta Path. microbiol. Scand. A*, **83**, 80.

TSUCHIDA, Y., ENDO, Y., URANO, Y. & ISHIDA, M. (1975) α-Fetoprotein in Yolk Sac Tumor. *Ann. N.Y. Acad. Sci.*, **259**, 221.

TSUCHIDA, Y., SAITO, S., ISHIDA, M., OHMI, K., URANO, Y., ENDO, Y. & ODA, T. (1973) Yolk Sac Tumor (Endodermal Sinus Tumor) and Alpha-fetoprotein. A Report of Three Cases. *Cancer, N.Y.*, **32**, 917.